# The efficacy and safety of intra-articular injection of triamcinolone acetonide versus triamcinolone hexacetonide for treatment of juvenile idiopathic arthritis

**DOI:** 10.1186/s12969-022-00666-x

**Published:** 2022-01-29

**Authors:** Shiri Rubin, Orly Ohana, Ori Goldberg, Orit Peled, Yulia Gendler, Zohar Habot-Wilner, Yoel Levinsky, Rotem Tal, Liora Harel, Gil Amarilyo

**Affiliations:** 1 Pediatric Hematology Oncology Division, Schneider Children’s Medical Center of Israel, Petach Tikva, Israel; 2 Sackler Faculty of Medicine, Tel Aviv University, Tel Aviv, Israel; 3 Department of Pediatrics C, Schneider Children’s Medical Center of Israel, Petach Tikva, Israel; 4 Pediatric Pulmonary Unit, Schneider Children’s Medical Center of Israel, Petach Tikva, Israel; 5 Department of Pharmacy, Schneider Children’s Medical Center of Israel, Petach Tikva, Israel; 6 The Department of Nursing, Ariel University, Ariel, Israel; 7 Division of Ophthalmology, Tel Aviv Sourasky Medical Center, Tel Aviv, Israel; 8 Department of Pediatrics B, Schneider Children’s Medical Center of Israel, Petach Tikva, Israel; 9 Pediatric Rheumatology Unit, Schneider Children’s Medical Center of Israel, Petach Tikva, Israel

**Keywords:** Juvenile idiopathic arthritis, Intra-articular corticosteroids, Triamcinolone

## Abstract

**Objectives:**

Juvenile idiopathic arthritis (JIA) is the most common childhood rheumatic disease. Intra-articular corticosteroids joint injection (IAJI), with triamcinolone hexacetonide (TH) or triamcinolone acetonide (TA), is an effective additional treatment for oligo and polyarticular JIA. Previous studies have shown the benefits of TH over TA; however, TA is still used in many pediatric rheumatology centers. Our unit has experience with both regimens, and therefore we aimed to compare the efficacy and safety of TA versus TH for JIA patients.

**Methods:**

Chart review of JIA patients who were randomly (based on drug availability) treated with TA or TH IAJI during 2010–2019. Primary outcomes for efficacy were defined as full recovery from arthritis one month after IAJI and a relapse rate of arthritis 3 months after IAJI. Primary outcome for safety was defined as the occurrence of adverse events (AEs) during the follow up period after IAJI.

**Results:**

Overall, 292 joints of 102 JIA patients were treated (138 TA/154 TH joints). Complete recovery after one month was documented in 107 (69.6%) of TA treated joints and 96 (69.5%) of TH treated joints (*P* = 0.232). However, rate of relapse after 3 months was significantly higher for TA treated joints (27 (20.1%) vs. 13 (8.8%), respectively, *P* < 0.01). No AEs were documented except minor scars at four joint injection sites.

**Conclusion:**

The recovery from arthritis was similar (~ 70%) with both regimens, however relapse rate was more than double in TA as compared to TH injected joints. These findings are important due to a contemporary shortage of TH in the US market.

## Introduction

Intra-articular corticosteroid joint injection (IAJI) is one of the most prescribed medications for oligoarticular JIA [1]. In accordance with current 2019 American College of Rheumatology (ACR) clinical guidelines, IAJI is recommended as adjunct therapy for oligarticular and polyarticular JIA, especially when prompt disease control is needed [2, 3]. Response to intraarticular glucocorticoids is usually good, and many patients with oligoarthritis enter complete remission [1]. Several steroid preparations with different pharmacological properties are available for intra-articular injections in children. The beneficial effects and its duration are affected by the pharmacological properties of the type of preparation. In general, compounds that are less soluble and absorbed more slowly maintain synovial levels for longer periods with prolonged effect, resulting in lower systemic glucocorticoid levels [4–7]. The longest acting IAJIs used in clinical practice are triamcinolone hexacetonide (TH) and triamcinolone acetonide (TA) [4, 5]. It has been shown that the choice of steroid preparation depends on a variety of considerations such as commercial availability and the institution where the rheumatologist was trained [8, 9]. Several studies comparing the effectiveness of TH and other steroid preparations, including TA in children with JIA, have demonstrated the superiority of TH in terms of longer duration of action [4, 9–13]. However, TA is still used in many pediatric rheumatology centers [14]. Our rheumatology unit has experience with both regimens based on the availability of these drugs, and therefore we aimed to compare the short- and long-term efficacy and safety of TA versus TH for patients with oligo and poly-articular JIA.

## Methods

### Study design and patients

This is a retrospective chart review. We reviewed medical charts of children who fulfilled the ILAR revised diagnostic criteria for persistent/extended oligoarticular or polyarticular JIA [15], and had been treated with IAJI injection at Schneider Children’s Medical Center’s rheumatology unit during 2010–2019. Patients who had other chronic rheumatological illness, previous IAJI treatments in the last 4 months, or previous severe adverse reactions to steroids were excluded from the study.

Our pediatric rheumatology unit used TA for IAJI in JIA patients until May 2016, when TA was replaced by TH in June 2016 due to its availability and financial considerations. An intra-articular injection was performed according to clinical decision by a pediatric rheumatologist using standard protocol: prepare the skin with an antiseptic solution, aspirate the joint fluid to ensure the proper positioning of the needle, and then inject TA or TH. We used recommended doses [1, 4, 14]. TH was used at a dose of 1 mg/kg (maximal dose of 40 mg) in knees, and 0.5 mg/kg (maximal dose of 20 mg) in ankles, elbows, and wrists. In smaller joints (wrist, midtarsal, and subtalar), 0.3 mg/kg (maximal dose of 10 mg) was injected. TA was used at a dose of 1–2 mg/kg (maximal dose of 80 mg) in knees, 0.5–1 mg/kg in ankles and elbows (maximal dose of 40 mg), and 0.3–0.5 mg/kg (maximal dose of 40 mg) in wrist, midtarsal, and subtalar joints. All children were sedated for the procedure. No local or intraarticular analgesics were used. The procedure was done without ultrasound guidance. Children were advised not to bear weight for 24 h, and not to run or jump for another 48 h.

### Clinical evaluation and outcomes

Clinical assessment of arthritis was performed (as part of a clinical follow-up) by a pediatric rheumatologist at baseline, during the injection procedure, at ~ 1 month and ~ 3 months after the procedure, and at each follow-up visit thereafter. Complete response was defined as the absence of arthritis, and partial response as an improvement of arthritis (decrease in swelling or joint tenderness and/or increased range of motion). No response was defined as no change or worsening of arthritis. Primary outcome for efficacy was defined as full recovery from arthritis 1 month post IAJI, and relapse rate of arthritis 3 months post IAJI was defined as flare of arthritis after achieving complete response. Primary outcome for safety was defined as the occurrence of adverse events (AEs) during the follow-up period after IAJI. The AEs severity was classified according to the OMERACT 8 drug safety workshop [16].

The study was approved by the Research Ethics Board of Rabin Medical Center (approval no. RMC-18-0057).

### Data analysis

The following parameters were analyzed: gender, age of disease onset, disease duration, JIA type, number of joints involved, type of joint injected, first/re-injection, and laboratory parameters at baseline (C-reactive protein (CRP), erythrocyte sedimentation rate (ESR), antinuclear antibodies (ANA) positive result (ANA ≥1:40 titer using Immunofluorescence exam), anti-CCP antibodies (in patients with polyarticular JIA ≥ 7 years of age)), and concomitant therapy with NSAID’s, methotrexate (MTX), or anti-tumor necrosis factor (TNFα) such as etanercept or adalimumab.

All results were expressed as mean and standard deviation (SD), median; and minimum and maximum, or frequency and percentage. The differences in patients’ characteristics between the treatment groups were analyzed using chi-square test. Independent samples t-test or Mann-Whitney U test were used when normal distribution was not justified. The response rates for both treatment groups were compared using chi-square. The efficacy of intraarticular TA or TH injection was analyzed using the Kaplan-Meyer test, estimating the time of arthritis flare over a period of 40 months; missing data were censored. The analyses were performed by using SPSS software, version 25 (IBM, Chicago, Ill, USA), tests were two-tailed, and *p* values< 0.05 were considered statistically significant.

## Results

The demographic, clinical, and laboratory parameters of both study groups are summarized in Table 1. Of 102 patients in our study, 73% were females. The mean age of disease onset was 4 years, and mean disease duration at the time of treatment 1 year. Most (83%) patients had persistent oligoarticular JIA, followed by extended oligoarticular and RF negative polyarticular JIA.

**Table 1:** Demographic, clinical and laboratory parameters of patients who received IAJI in both treatment groups

	TA	TH	*P* value
Number of patients	51	51	
Number of joints, n(%)	138 (47.3%)	154 (52.7%)	0.44
Female, n(%)	37 (72.5%)	38 (74.5%)	0.50
Male, n(%)	14 (27.5%)	13 (25.5%)	
Age at onset (yr) mean ± SD median	4.39 ± 3.42 2.97	4.18 ± 3.16 3.01	0.76
Disease duration per joint, yr, mean (range)	0.9 (0.086–6.84)	1.14 (0.07–9.30)	0.331
JIA subtype, n(%)			
Persistent oligoarticular	42 (82.4%)	43 (84.3%)	0.85
Extended oligoarticular	6 (11.8%)	5 (9.8%)	
RF negative polyarticular	3 (5.9%)	3 (5.9%)	
Laboratory parameters at baseline:			
CRP (mg/dL) mean ± S.D. median	(±1.3) 0.8	1.3 (±1.2) 0.94	0.47
ESR (mm/hr) mean ± S.D. median	29.8 (±17.3) 28.5	28.9 (±17.1) 24.5	0.83
ANA positive (> 1:40)	26 (51%)	31 (±60.8%)	0.27
Type of joint injected, n(%)			
Knee	85 (61.6%)	95 (61.7%)	0.45
Ankle	33 (23.9%)	40 (26%)	
Wrist	6 (4.3%)	11 (7.1%)	
Elbow	11 (8%)	7 (4.5%)	
Other (fingers& toes etc.)	3 (2.2%)	1 (0.6%)	
Number of joints first injected, n(%)	98 (71%)	100 (64.9%)	0.162
Number of joints re-injected, n(%)	40 (29%)	54 (35.1%)	
Systemic treatment (per injection), n(%)			
NSAID’s TX during injection	106 (76.8%)	125 (81.2%)	0.220
NSAID’s after injection	51 (37%)	33 (21.4%)	0.003^*^
MTX during injection	14 (10.1%)	14 (9.1%)	0.457
MTX after injection	53 (38.4%)	49 (31.8%)	0.146
Anti TNF**α** during injection	3 (2.2%)	3 (1.9%)	0.605
Anti TNF**α** after injection	20 (14.5%)	31 (20.1%)	0.133

*TA* Triamcinolone acetonide, *TH* Triamcinolone hexacetonide, *RF* Rheumatoid Factor, *NSAID’S* Non-Steroidal Anti-inflammatory drugs, *MTX* Methotrexate

^*^Significance: *P* < 0.05

Of 292 joints injected the majority were in knees (61.6%) and ankles (25%), with 198 (67.8%) joints injected for the first time. A total of 94 (32.2%) joints were re-injected, 25 of them first with TA and when flared, re-injected with TH.

Both groups were comparable for age of onset, disease duration, gender, mean number of joints per patient, type of joint injected, JIA subtype, laboratory parameters, and concurrent systemic therapy with MTX and anti-TNFα during and after IAJI. The rate of NSAID’s given after IAJI was significantly higher in the TA group. Mean follow-up duration was 21.9 (range 4–95) months.

One patient with polyarticular JIA was older than 7 years old. His anti-CCP antibodies level was normal.

Patients with overall twenty eight joints (14 in each treatment group) were additionally treated with systemic MTX during the time of injection procedure. In 6/28 joints, the therapy was started 6–12 weeks prior the IAJI. Among them 2 joints had not relapsed in the study period. Patients with overall 6 joints (3 in each treatment group) were treated with anti TNFα during the time of IAJI procedure. in 3/6 joints the therapy was started 6–12 weeks prior the IAJI (18 months for 2 joints and 4 months for 1 joints). Among them 2 joints had not relapsed in the study period and 1 joint had relapsed 7 months after the injection.

Table 2 summarizes the differences in response rate for both treatment groups. Response rate at one month after injection was similar in both groups. Complete response, defined as absence of arthritis, was also similar in both groups (69.6% vs. 69.5%, respectively, *P* = 0.232). Partial response, defined as an improvement of arthritis, was seen in 23.2 and 16.9% of patients in the TA and TH groups, respectively, *P* = 0.232. No response or worsening was seen in 7.2 and 11.7%, respectively, *P* = 0.232. As seen in Table 2, most joints maintained complete response at 3 months after injection, however relapse rate after 3 months was significantly higher in the TA treated joints (20.1% vs. 8.8%, respectively, *P* = 0.018).

**Table 2:** Comparison of response rate between JIA patients in both treatment groups

	Response at 1 month			Response at 3 months^&^		
	TA	TH	***p***-value	TA	TH	***p***-value
Complete response	96 (69.6%)	107 (69.5%)	0.232	93 (69.4%)	107 (72.3%)	0.018^*^
Partial response	32 (23.2%)	26 (16.9%)		8 (6.0%)	18 (12.2%)	
No response	10 (7.2%)	18 (11.7%)		6 (4.5%)	10 (6.8%)	
Relapse	NA	NA		27 (20.1%)	13 (8.8%)	

*TA* Triamcinolone acetonide, *TH* Triamcinolone hexacetonide

At 3 months after IAJI, no information was available for four joints in the TA group and three joints in the TH group

^*^Significance: *P* < 0.05

**Fig. 1:**
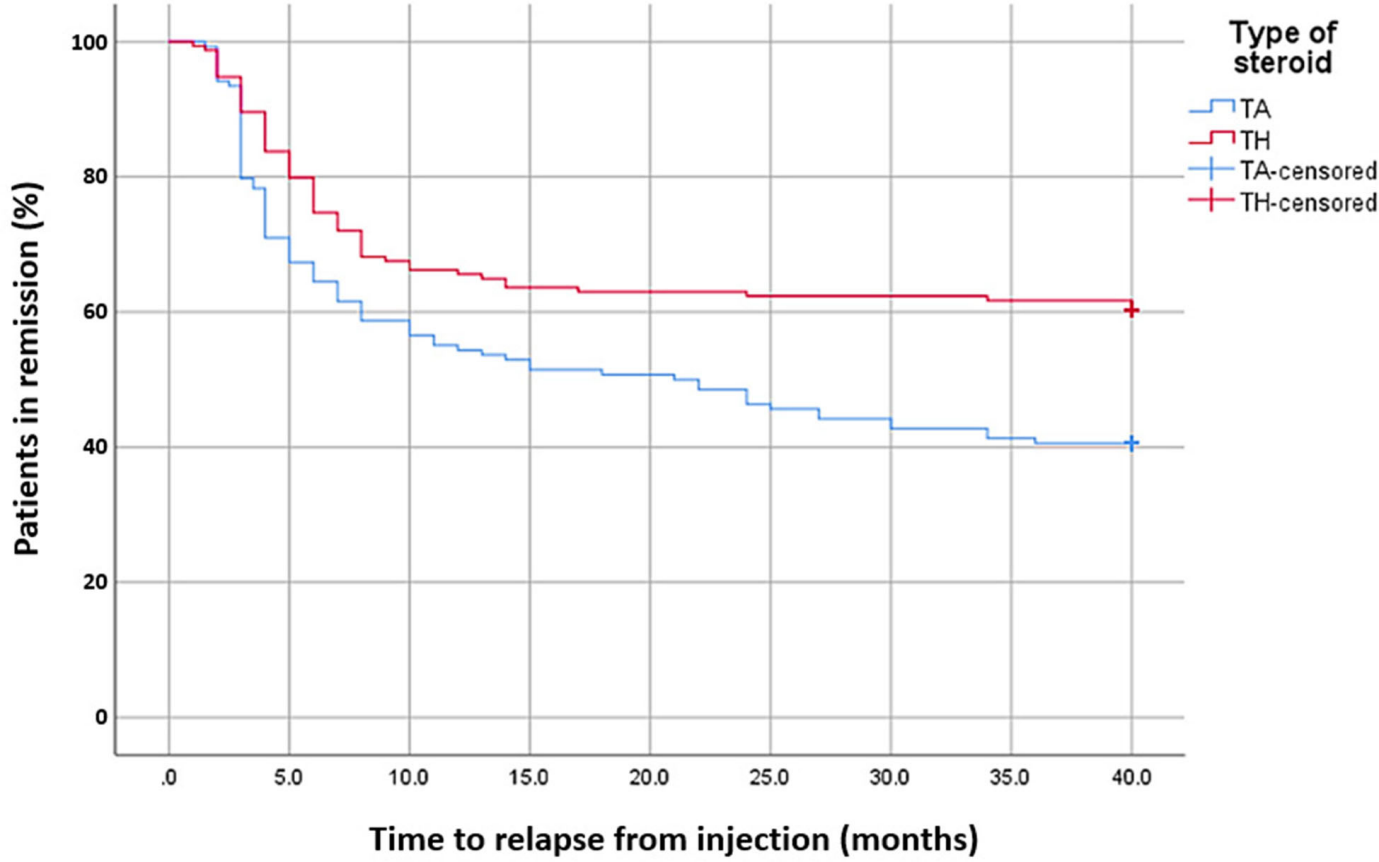
Kaplan-Meyer analysis of relapse rates in both treatment groups (*P* = 0.02, log rank test) TA - Triamcinolone acetonide; TH - Triamcinolone hexacetonide

Kaplan-Meyer analysis, which compared the efficacy of intraarticular TA or TH injection over a period of 40 months (Fig. 1), showed a significant higher relapse rate in TA vs. TH treated joints from 3 months after injection and throughout the follow-up period (*p* < 0.02). Logistic regression model showed the odds ratio for relapse was 2.24 (95% CI 1.39–3.58, *p* = 0.001) when using TA for IAJI.

Mild adverse reactions, such as skin atrophy or hypopigmentation at the injection site, were seen in only 4/292 (1.4%) of injected joints, for two joints in each group. No other complications, such as joint infection or chemical synovitis, were noted.

## Discussion

Although studies show better long-term efficacy of TH vs. TA, there is a paucity of data regarding the short-term efficacy of both drugs [9, 11–13]. Therefore, by counting on personal experience, the treating physician may be misled by assuming that TA and TH have overall similar efficacy. Our study shows that both TA and TH have similar efficacy 1 month after IAJI (i.e., induction of remission). However, after 3 months, the relapse rate of arthritis was significantly lower in the TH group as compared to the TA group (8.8% vs. 20.1%, respectively, *P* = 0.018). Moreover, this trend was sustained during the 40-month follow up with an odds ratio to relapse more than double [2.24 (CI 1.39–3.58)] in TA injected joints.

As previously noted, earlier studies have addressed the long-term efficacy of TH vs. TA in JIA patients who received IAJI. Zulian et al. noted, in a prospective study, a significantly higher rate of response with TH than with TA at 6 months, 12 months, and 24 months (60% vs. 33.3%, respectively) [12]. Although not mentioned directly in their manuscript, a Kaplan Meier curve showed similar efficacy of TH and TA at 1 month after injection. The same group later showed similar long-term results in a blinded prospective study of symmetrical IAJI, where one joint was treated with TH and the other with TA [9]. In addition, it was shown that even when TA was given at a dose twice that of TH (2 mg/kg vs 1 mg/kg accordingly) in symmetrical involved joints, TH was more effective than TA during short- and long-term follow up [9]. Another study by Eberhard et al. compared time to relapse in a follow up of 15 months after TH or TA injection of 227 joints. At 6 months, they showed a response rate of 76% vs. 56%, respectively. However, the effect of TA, but not TH, appeared to subsequently wane, with a response rate of approximately 50% in the TH group vs. only 21% in the TA group after 12 months [13]. In addition, they found the hazard ratio attributed to the injection type was 1.8 (95% CI 1.05, 3.08). Unlike our study and the Zulian group studies [9, 12], their TA group had a higher relapse rate starting in the first month after injection.

In our study, patients with overall 34 joints were treated additionally with systemic therapy of either MTX or TNFα during the IAJI time. However, groups were balanced (17 patients in TH and 17 in TA). Moreover, in sensitivity analysis, results were comparable with the study findings.

Interestingly, patients with TH injected joints were more likely to be treated with anti TNFα as compared to patient treated with TA (31 vs. 20, respectively), presumably due to better TNFα availability. None the less, these differences were not significant (*p* = 0.133).

Of note, no correlation was found between disease duration to the outcome of the first injection in both treatment groups.

IAJI is an important adjunct therapy in oligo-articular and polyarticular JIA that can serve as optimal initial therapy in oligoarticular JIA. The intraarticular approach delivers a high concentration of corticosteroid to the synovial fluid of the inflamed joint, there they influence different immunological functions, eventually reducing the migration of leukocytes into the joint [5, 7].

TH and TA are the most commonly used long-acting steroids for intra-articular injection. TH (molecular weight 532.66) differs from TA (molecular weight 434.49) by an alteration of one side chain, which presumably makes TH less water soluble as compared with TA. Compounds with lower water solubility maintain effective synovial levels, thus providing longer duration of effectiveness within the peripheral joint space [5, 7]. Additionally, compounds with lower solubility are absorbed more slowly, resulting in lower peak plasma minimizing systemic effects such as adrenal suppression [17, 18]. Indeed, pharmacokinetic studies show that due to its lower solubility, TH is absorbed more slowly than TA, maintaining synovial levels for a longer period (6 and 3.2–4.3 days, respectively) and creating lower systemic glucocorticoid levels [7]. This may explain the similar very short-term efficacy of both drugs vs. the higher relapse rate that was found in TA compared to TH injections. Furthermore, pharmacokinetic studies have shown that 40 mg of TA is equivalent to 20 mg of TH with regard to biological effect [7]. Many centers are still using TA in their clinical practice, mostly due to financial considerations and drug availability. Moreover, due to lack of practice guidelines, there are considerable variations of the dosages used in pediatric rheumatology centers around the globe for both regimens [14]. The price difference between both regimens is negligible (around 1.5 USD/mg for TH vs. 0.5 USD/mg for TA). Our rheumatology unit has experience with both steroid preparations and the decision to use a specific formulation is generally based on availability of either drug in out hospital. This provided an opportunity to compare the short- and long-term effectiveness of TA compared to TH.

Although IAJI can be an effective and safe line of treatment, repeated intra-articular steroid injections may predispose to charcot’s arthropathy as a potential side effect [19]. In our study, adverse events were infrequent and mild, including skin atrophy and hypopigmentation at the injection site in only four (1.4%) joints, two in each group. This rate is comparable with the incidence reported in the literature ranging from 2 to 8% [9, 11, 14, 20].

Our study has several limitations. First, it is a retrospective study. Second, although this was the largest cohort reported to date, the number of injected joints was relatively small and not sufficiently powered. Third, our study is composed of a heterogeneous group of patients including a large portion with re-injected joints and patients treated with MTX or biological therapy during the study period. Yet, subgroup sensitivity analysis has not demonstrated significant differences among groups. Fourth, validated scoring systems such as Juvenile Arthritis Disease Activity Score (JADAS), were lacking.

## Conclusions

The recovery from arthritis was similar (~ 70%) with both regimens, however relapse rate (defined as flare of arthritis after achieving complete response) was more than double in TA as compared to TH injected joints. These findings are especially important due to the contemporary shortage of TH in the US market, and usage of TA in many pediatric rheumatology centers for IAJI around the globe.

## Abbreviations

JIA: Juvenile idiopathic arthritis
IAJI: Intra-articular corticosteroids joint injection
TH: Triamcinolone hexacetonide
TA: Triamcinolone acetonide
AE: Adverse events
ACR: American College of Rheumatology
CRP: C-reactive protein
ESR: Erythrocyte sedimentation rate
ANA: Antinuclear antibodies
NSAID’s: Non-steroidal anti-inflammatory drugs
MTX: Methotrexate
TNF: Tumor necrosis factor

## Declarations

The study was approved by the Research Ethics Board of Rabin Medical Center (approval no. RMC-18-0057).
